# Research Progress on the Wear Resistance of Key Components in Agricultural Machinery

**DOI:** 10.3390/ma16247646

**Published:** 2023-12-14

**Authors:** Ying Wang, Dong Li, Cheng Nie, Pan Gong, Junsheng Yang, Zhigang Hu, Bin Li, Ming Ma

**Affiliations:** 1 School of Mechanical Engineering, Wuhan Polytechnic University, Wuhan 430023, China; ywang0515@163.com (Y.W.); niecheng1031@163.com (C.N.); yangjunsheng2008@163.com (J.Y.); hzg@whpu.edu.cn (Z.H.); libin_027@126.com (B.L.); maming@whpu.edu.cn (M.M.); 2 State Key Laboratory of Materials Processing and Die & Mould Technology, School of Materials Science and Engineering, Huazhong University of Science and Technology, 1037 Luoyu Road, Wuhan 430074, China; pangong@hust.edu.cn

**Keywords:** agricultural machinery, key components, wear resistance, research progress

## Abstract

Agricultural mechanization is crucial in enhancing production efficiency, alleviating labor demands, reducing costs, improving agricultural product quality, and promoting sustainable development. However, wear and tear are inevitable when using agricultural machinery. The failure of critical wear-resistant parts is responsible for over 50% of rural machinery breakdowns. For instance, a domestic combine harvester typically only operates trouble-free for 20 to 30 h, and the service life of a rotary plow knife is approximately 80 h. Investigating the wear performance of key farm machinery components reinforces machinery design and maintenance strategies, extends machinery lifespans, enhances agricultural production efficiency, and advances agrarian sustainability. This paper provides a comprehensive overview of the latest research on the wear resistance of crucial agricultural machinery components. It delves into the factors influencing the wear resistance of these components and explores current effective measures to address wear-related issues. Additionally, it also summarizes the challenges and opportunities in researching the wear performance of key components in agricultural machinery and future development directions.

## 1. Introduction

Traditional farming methods rely on inefficient human and animal labor and are easily affected by factors like climate and season [1,2]. In contrast, agricultural mechanization replaces traditional labor with advanced machinery, enhancing planting, harvesting, and crop handling efficiency. It reduces labor work and time while adapting to diverse environments and seasons. Furthermore, agricultural mechanization improves the quality and variety of agricultural products [3]. Advanced agricultural machinery allows for the precise control of soil moisture, temperature, and nutrient supply, thereby enhancing crop growth and yield [4]. However, agricultural mechanics operate in open-air environments, are often exposed to wet and corrosive conditions, and come into contact with soil, gravel, crop stalks, and roots. As a result, they endure significant wear and tear and vibration and shock loads during operation [5]. Therefore, agricultural machinery parts must exhibit excellent wear resistance, besides requiring adequate strength, rigidity, and toughness. Research on rural machinery wear opposition spans various fields, including mechanical engineering [6], materials science [7], and surface-strengthening treatments [8]. In the future, with the intelligent [9] and precise and green development of agricultural machinery [10], research on the wear resistance of agricultural machinery will face new challenges and opportunities. Developing and applying new materials, developing intelligent monitoring and maintenance technology, improving precision manufacturing, and meeting green environmental protection requirements will all become essential directions for future research on the wear resistance of agricultural machinery [11,12].

This article provides an overview of the latest research status on the wear resistance of key components in agricultural machinery, focusing on the study of wear mechanisms, research on wear-resistant materials, and the application of new wear-resistant technologies. It examines the factors that affect the wear resistance of crucial components in agricultural machinery through material selection and its microstructural changes, manufacturing processes, and the operational conditions of agricultural machinery. Additionally, it systematically summarizes and analyzes methods to improve the wear resistance of these key components, including optimizing the design of mechanical parts, using wear-resistant materials, and applying coatings to their surfaces. It also summarizes the challenges and opportunities in researching the wear performance of key components in agricultural machinery and future development directions.

## 2. Research Status on the Wear Resistance of Key Agricultural Machinery Components

Among the various parts and components of agricultural machinery, key elements collectively include plowshares, harrow blades, rotary tiller and harvester blades, no-till planter furrowing discs, crusher liners, tractor tracks, transmission and conveyor belts, diesel engine crankshafts, and other parts. Their performance during use fundamentally determines the overall quality of agricultural machinery [13]. Currently, research on the wear resistance of agricultural machinery primarily focuses on three aspects: the investigation of wear mechanisms [14], the examination of wear-resistant materials [15], and research on the application of new wear-resistant technology [16].

### 2.1. Research on Wear Mechanisms

Agricultural machinery is an essential tool in agricultural production, and mechanical wear is a crucial factor influencing its performance and lifespan. Research on the wear mechanism of agricultural machinery can offer a scientific foundation for prolonging the service life of agricultural machinery and enhancing agricultural production efficiency.

Scholars have investigated the formation mechanism and influencing factors of agricultural machinery wear through experiments and theoretical modeling, primarily encompassing abrasive, adhesive, and corrosive wear [17]. In 2020, Borak K et al. [18] examined abrasive particles’ shape alteration and wear capacity in various Ukrainian soils under rotary tillage and sowing field tests. They determined a range shape of factors of abrasive particles in Ukrainian soils. The findings revealed that when the shape factor of soil abrasive particles in the tilled layer was less than 11.43, their wear decreased by 47.71% compared to the untilled layer. This confirms the significant impact of soil abrasive particles’ hardness, shape, and size on abrasive wear. Table 1 summarizes the formation mechanism and influencing factors of different types of agricultural machinery wear. Table 2 outlines the forms and causes of wear failure in agricultural machinery components.

Research on wear mechanisms in agricultural machinery also emphasizes the development of methods for predicting and evaluating wear. Through modeling and experimental validation, scholars can predict agricultural machinery wear under varying working conditions and consider its service life and maintenance cycle. Bedolla PO et al. [19] have devised integrated experimental and numerical simulation methods based on Archard’s time- and space-resolved wear equations to assess and forecast the impact of soil abrasive wear on rotary tiller blade components. These methods enable qualitative and quantitative assessments of rotary tiller blades’ soil abrasive wear mechanism. Their accuracy and practicality have been demonstrated on disc harrow tines. Such methods hold significance in optimizing machinery design and maintenance strategies to mitigate the risk of wear-induced failures and production downtime.

Current research on the wear mechanism of agricultural machinery has made notable advancements. However, it still faces challenges and unresolved issues warranting further in-depth investigations. For instance, addressing how to mitigate mechanical wear in intricate working conditions and how to meet durability demands, along with selecting suitable materials and lubrication techniques to minimize wear, remains crucial.

**Table 1:** Formation mechanism and influencing factors of different types of wear in agricultural machinery.

Type of Wear	Formation Mechanism	Influencing Factors	References
Abrasive wear	Agricultural machinery in the soil works partly on the surface of high-hardness abrasive particles; when mechanical features are in motion and in contact with these abrasive particles, wear and tear occurs.	Size and hardness of abrasive grains, surface hardness of machine parts and materials	[20]
Adhesive wear	In agricultural machinery, surface wear occurs as a result of mechanical parts’ surfaces adhering at high temperatures and pressures and then peeling off with relative motion.	Surface roughness, material selection, lubrication	[21]
Corrosive wear	Corrosive wear is usually caused by agricultural machinery operating in wet or corrosive environments, resulting in the material’s surface being subjected to corrosive media. This can cause the material’s surface to corrode, dissolve, or lose its protective layer, ultimately leading to wear.	Nature, temperature, concentration of corrosive medium, corrosion resistance of materials	[22]

**Table 2:** Types and causes of wear failure in agricultural machinery parts and components.

Part Name	Form of Wear	Reasons for Wear Failure	Reference
Tractor transmission gears	Abrasive wear	The transmission gear assembly of a tractor is generally made of a steel material, which often leads to abrasive wear due to metal collisions over long periods and the ingress of particulate matter.	[23]
Rotary plow knives	Abrasive wear	Rotary tiller blades are used to deeply till and turn the land to improve the looseness of the land, but during use, rotary tiller blades are prone to abrasive wear due to contact with the land surface.	[24]
Water pump impeller	Adhesive wear	Irrigation water contains impurities such as dirt and sand, which can lead to adhesion between the impeller and the water pipe, thus accelerating the wear and tear of the impeller.	[25]
Straw crusher hammer blades	Adhesive wear	Straw contains impurities such as soil and sand, which will lead to adhesion between the hammer blade and the screen, thus accelerating the wear of the hammer blade.	[26]
Agricultural sprayer nozzles	Corrosive wear	Due to the frequent spraying of aqueous solutions containing chemical pesticides and fertilizers, the nozzle’s inner wall erodes, leading to wear and tear.	[27]

### 2.2. Research on Wear-Resistant Material

The choice of wear-resistant materials for agricultural machinery directly impacts the service life and efficiency of the machinery. In the current state of research, selecting wear-resistant materials for agricultural machinery is a multifaceted issue that necessitates a thorough assessment of material performance, cost, and efficiency.

**Figure 1:**
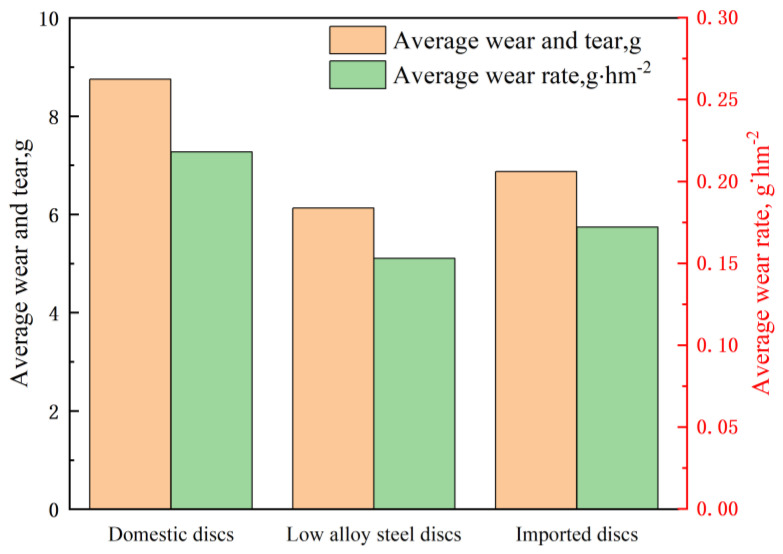
Comparison of wear effects of three types of furrowing discs in field trials.

When selecting materials, it is essential to consider factors such as the working environment, working conditions, and the intensity of mechanical parts. Material properties like wear resistance, strength, toughness, and cost must also be considered. Current research indicates that commonly used wear-resistant materials in agricultural machinery include cast iron, high-manganese steel, and high-chromium alloys [28]. Scholars have studied these materials’ microstructure, chemical composition, and heat treatment processes to enhance their wear-resistant properties [29]. Moreover, considering the specific working conditions of agricultural machinery, researchers have developed wear-resistant ceramic composites (such as ZrB2-based materials) for use in plow heads and harrow heads [30]. These wear-resistant ceramic composites offer advantages such as lightweight, high strength, and excellent wear resistance, meeting the requirements for agricultural machinery in terms of service life and efficiency [31]. Xing Renjie et al. [32] statistically field tested data of a no-till planter core component, a furrow disc. Figure 1 shows that the average wear rate is more significant for imported furrow discs than for lower-alloy discs. In contrast, the domestic disc (65 Mn steel) has the worst wear resistance. The primary reason for this is that a strength of 65 Mn is comparatively low, making it susceptible to plastic deformation and fracture, which hinders its ability to withstand impacts and twisting forces effectively. Moreover, 65 Mn steel exhibits poor corrosion resistance, quickly succumbing to oxidation and corrosion in the surrounding environment. These factors diminish the material’s overall service life, making it unsuitable for prolonged exposure to humid and corrosive furrowing conditions. Furthermore, 65 Mn steel is prone to thermal softening at high temperatures, reducing its original hardness and strength. This shows that 65 Mn steel is not the best material for furrowing discs.

Advancements in new materials and improvements in conventional materials have enhanced the wear resistance of critical components in agricultural machinery. Moreover, surface-strengthening treatments have emerged as a prominent research focus. Techniques like surface coating and heat treatment technology effectively enhance the wear-resistant properties of key agricultural machinery components, thus extending their service lives.

### 2.3. Research on the Application of New Wear-Resistant Technology

In recent years, with the continuous advancement of science and technology, nano-lubrication technology and computer-assisted techniques have played a crucial role in researching wear resistance in agricultural machinery. They have provided new ideas and methods for improving the wear-resistance performance of agricultural machinery.

The application of lubrication technology in studying agricultural machinery wear resistance has seen significant advancements. Conventional lubricants, like mineral lubricants, suffer from poor oxidative stability and considerable viscosity changes with temperature, which impose certain limitations on enhancing agricultural machinery wear resistance [33]. Scholars have initiated investigations into novel nano-lubricants, utilizing nano-silicon and nano-oxides as lubricants. These nano-lubricants can create a uniform and stable nano-lubrication film, reducing frictional wear in agricultural machinery and improving wear-resistant performance [34]. Moreover, nano-lubrication technology can potentially reduce energy consumption and enhance agricultural machinery efficiency, presenting a promising avenue for its application.

Using computer-aided technology to study agricultural machinery wear resistance has gained increasing prominence [35]. Computer simulation technology can simulate and predict the wear process of agricultural machinery components, offering a crucial foundation for material improvements and design enhancements. For example, CucinottaF et al. [36] employed a structured blue-light 3D scanner to scan plowshares before and after field cultivation tests. They analyzed and compared the wear patterns among four plowshares, obtaining contour parameters for the plowshares and cutting edges. Subsequently, they employed computer-aided design technology to optimize the plowshare’s structure and shape to reduce wear friction and enhance wear resistance.

Applying lubrication technology and computer-aided technology in studying agricultural machinery wear resistance introduces fresh ideas and methods for enhancing agricultural machinery wear resistance. Lubrication technology employs nano-lubricants to establish a consistent and stable nano-lubrication film. In contrast, computer-aided technology offers technical support for optimizing agricultural machinery structure through wear process simulation and prediction. These technologies’ ongoing enhancement and advancement create new opportunities to improve rural machinery wear resistance.

## 3. Factors Affecting the Wear Resistance of Key Components in Agricultural Machinery

In agricultural machinery’s service environment, wear resistance has become a crucial factor limiting its performance and lifespan due to harsh working conditions and frequent operations [37]. Comprehending the factors that influence agricultural machinery wear resistance is imperative for enhancing machinery reliability, reducing maintenance requirements, and meeting agricultural production needs effectively. These factors encompass material selection and their micro-structural changes, the machining process, and the service environment of agricultural machinery.

### 3.1. Material Selection and Microstructure Evolution

To enhance the wear resistance of mechanical parts, scholars must consider not only the fundamental physical and chemical properties of materials but also delve into the materials’ microstructure. A material’s microstructure encompasses its grain structure, phase distribution, grain boundaries, dislocations, and other microscopic characteristics. Changes in these features can significantly impact a material’s mechanical properties and wear resistance. By choosing appropriate materials and employing suitable heat treatment processes, material microstructure can be controlled, optimizing the wear resistance of key components in agricultural machinery.

**Figure 2:**
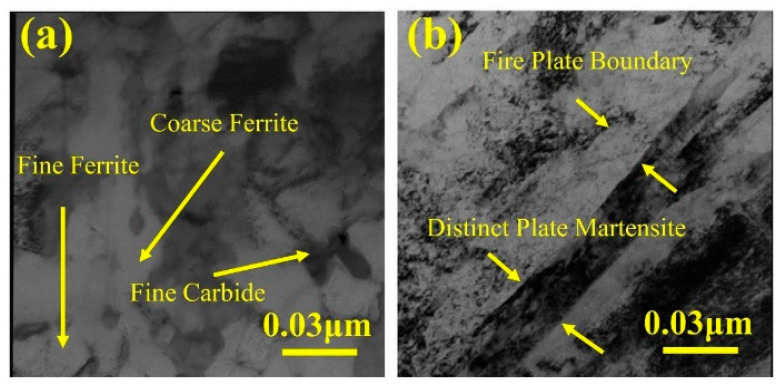
M50 microstructure before abrasion [39]: (**a**) before quenching treatment and (**b**) after quenching treatment.

**Figure 3:**
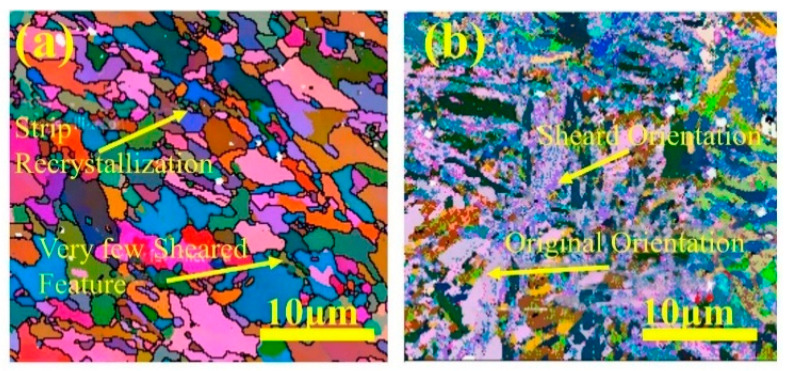
M50 EBSD measurements [39]: (**a**) before wear and (**b**) after wear.

M50 bearing steel is a high-performance specialty steel frequently used to fabricate high-speed rotating mechanical components such as bearings, gears, and drive shafts in agricultural machinery [38]. Mukhopadhyay et al. [39] used abrasive testing equipment (ASTM G 65-85) and applied 13 kg load to carry out wear test on M50 bearing steel before and after quenching treatment. They employed SEM and EBSD to examine the microstructure of the deformation zone during wear. Figure 2 displays the results, indicating that the microstructure of M50 bearing steel predominantly consists of ferrite and martensite before the wear test. In Figure 3, the martensite slats exhibit significant shear features after wear, while the residual austenite phase appears white. The SEM observations revealed numerous cratered shear components on the martensite slats. This phenomenon can be explained by the increased toughness of martensite due to softening and dynamic recrystallization, resulting in reduced tendencies for microcrack development, eliminating stress concentration regions, and phase refinement. The abundant shear features and dynamic recrystallization phenomena within the martensitic structure contribute to its superior wear resistance to the ferritic structure.

**Figure 4:**
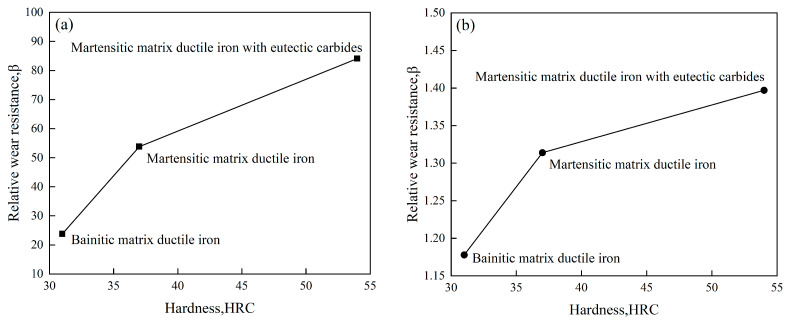
Wear resistance versus hardness for different matrix ductile cast irons in the pin-on-disc and three-body abrasion tests: (**a**) pin-on-disc abrasion test and (**b**) three-body abrasion test.

**Figure 5:**
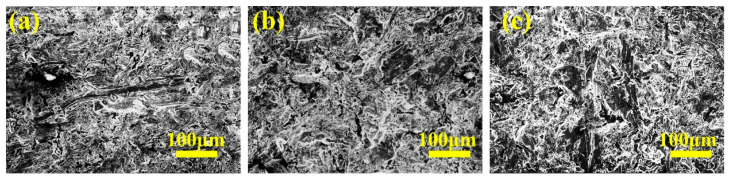
SEM morphology after wear [42]: (**a**) martensitic ductile iron, (**b**) bainitic ductile iron, and (**c**) martensitic diffuse eutectic carbide ductile iron.

Plow bodies and blades in agricultural machinery are essential for cultivation and land preparation, demanding excellent wear and corrosion resistance. Due to its cost-effectiveness, wide availability, and good wear resistance, cast iron is often chosen as the ideal material for manufacturing these components [40,41]. Lu et al. [42] investigated the abrasive wear behavior of ductile cast iron featuring three distinct matrix tissues using pin–disc and three-body wear tests. Their findings revealed that the abrasion resistance of ductile cast iron under both pin-on-disc and three-body abrasion conditions displayed an approximately linear relationship with hardness, as depicted in Figure 4. Furthermore, Figure 5a illustrates that the wear surface of the martensitic matrix exhibited long-distance micro-cutting and abrasive embedment within the matrix. In contrast, Figure 5b displays the wear surface of the bainite matrix, characterized by numerous deep spalling pits. Lastly, Figure 5c shows the wear morphology of martensitic matrix ductile iron with eutectic carbides, which is dominated by short-distance micro-cutting and shallow spalling pits. These observations suggest that the martensitic matrix and martensitic matrix ductile iron with eutectic carbides possess the highest wear resistance, followed by the bainitic matrix.

**Figure 6:**
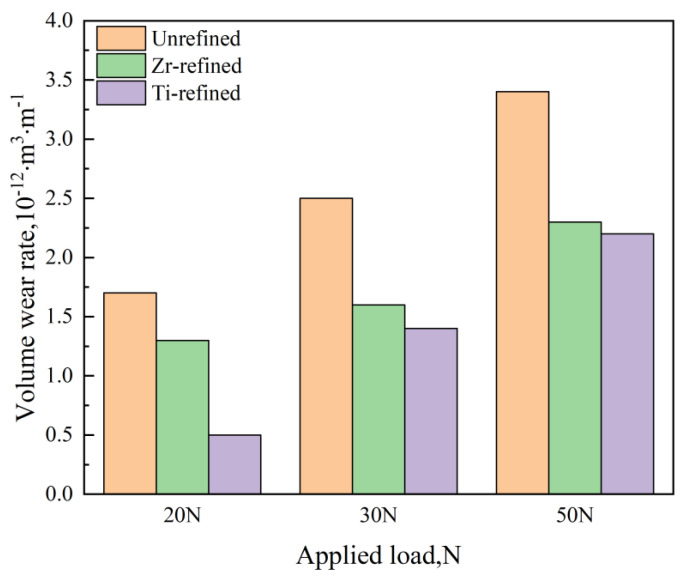
7042 Aluminum alloy wear rate.

**Figure 7:**
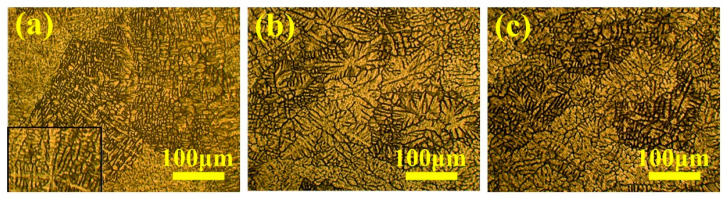
7042 Aluminum alloy microstructures [46] (**a**) without addition, (**b**) with Zr refiner, and (**c**) with Ti refiner.

Aluminum alloys are favored for their low density and excellent corrosion resistance. It is a common choice for crafting lightweight components in agricultural machinery, such as aluminum alloy beams and frames. However, they have limited wear resistance and struggle to achieve self-lubrication on their surfaces, hindering their widespread use in agricultural machinery [43,44]. Investigations have revealed that failures of aluminum alloy parts in agricultural machinery often originate from the surface, with surface friction and wear accounting for approximately 80% of such losses [45]. Farahani et al. [46] researched the impact of Zr and Ti refining agents on the microstructure and wear properties of a 7042 Aluminum Alloy. The research results show that, as depicted in Figure 6, the wear rate of the 7042 Aluminum Alloy increased with the applied load after Zr and Ti refinement. Notably, the grain-refined aluminum alloy exhibited less wear than the unrefined aluminum alloy. This is attributed to the crucial role played by grain size in material wear resistance, as demonstrated in Figure 7. The refined aluminum alloy’s structure transformed from coarse columnar α-Al dendrites to fine equiaxed α-Al dendrites, with fine α-Al dendrites reducing the grain boundary area and consequently lowering the material’s wear rate, thus enhancing its wear resistance.

The microstructure of a material can be controlled through processes like heat treatment, alloying, and machining to enhance its hardness, strength, and wear resistance. For instance, adjusting its grain size and distribution can increase a material’s hardness and improve its wear resistance. In addition, alloying can introduce harder elements such as carbides, nitrides, and hard phases to increase the wear resistance of a material.

### 3.2. Machining Process

Processing technology is a pivotal factor influencing the wear resistance of agricultural machinery and exerting a profound influence on mechanical components’ material properties and structural characteristics. Precise machining processes can enhance material surfaces’ finish and precision, diminish uneven stress distributions, and consequently enhance the anti-wear performance of mechanical parts. Simultaneously, suitable heat treatments can elevate a material’s hardness, wear resistance, and corrosion resistance.

**Figure 8:**
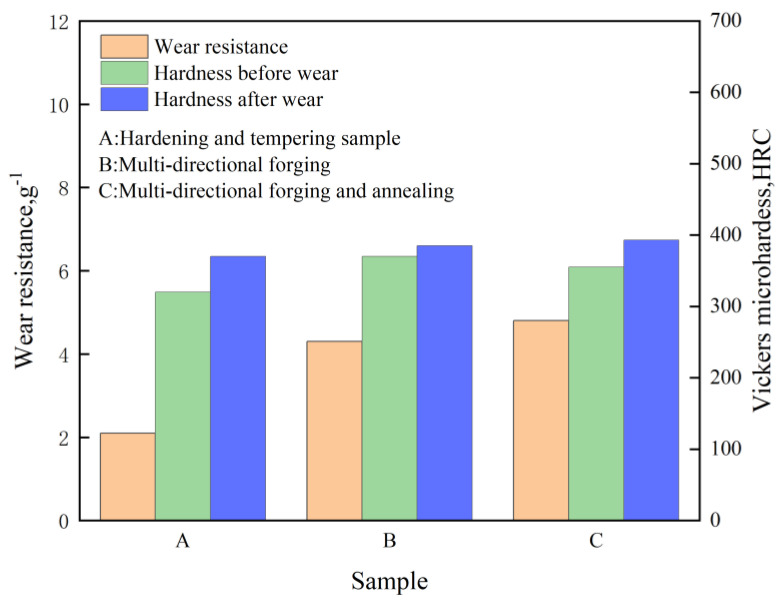
Changes in wear resistance and hardness of medium-carbon low-alloy steel before and after abrasion.

**Figure 9:**
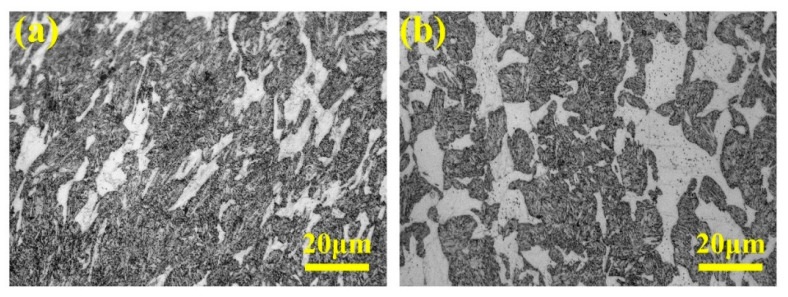
Cross-sectional SEM images [47]: (**a**) multidirectional forging; (**b**) multidirectional forging and heat treatment.

Liu et al. [47] conducted multidirectional forging and annealing on medium-carbon low-alloy steel to investigate the wear resistance of the specimens. As illustrated in Figure 8, the wear resistance increased by approximately 80% compared to the original samples after multidirectional forging. Multidirectional forging had a notable impact on abrasive wear properties, particularly affecting the tensile strength and hardness of the specimens subjected to multidirectional forging and annealing, which showed a slight decrease. However, the wear resistance increased by around 120% compared to the original samples. The critical factor behind this improvement lies in the refined ferrite grains in the multidirectional forging specimens, as depicted in Figure 9a. The reduced grain size decreases the grain boundary area, lowering the material’s wear rate [48]. Additionally, after multidirectional forging and annealing treatment, a substantial number of ultrafine carbide particles are dispersed throughout the grains, as shown in Figure 9b. This distribution aids in accumulating dislocations, enhances dislocation storage capacity, and prevents dislocation slippage, thus promoting work-hardening and ultimately improving plasticity. The wear resistance of each specimen is further improved.

**Figure 10:**
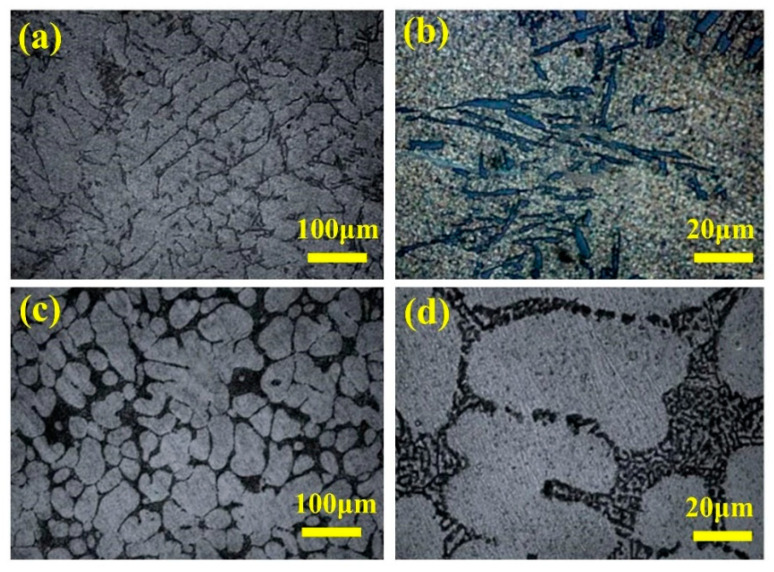
LM 25 SEM [49]: (**a**,**b**) conventional casting process; (**c**,**d**) modified casting process with Sr modifier.

**Figure 11:**
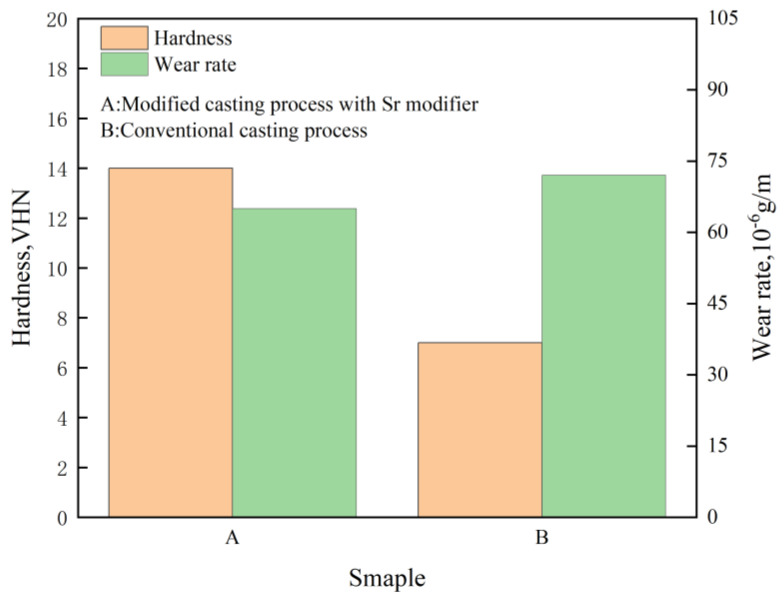
Variation in hardness and wear rate of LM 25 alloy.

Manani et al. [49] conducted a study to explore the impact of Sr densification, both with and without it, during the casting process on the wear resistance of an LM 25 alloy. The findings revealed that, as depicted in Figure 10, conventional and modified casting processes yielded alloys comprising α-Al dendrites and acicular eutectic Si particles. However, the alloys from the modified casting process featured smaller and more uniformly distributed modified eutectic Si particles. Figure 11 illustrates that when Sr densification was applied during the casting process, the resulting microstructure alteration led to a 53% reduction in the wear rate compared to that of a conventional casted alloy.

Forging and heat treatment, along with casting, constitute the two primary processing methods in the fabrication of agricultural machinery parts, jointly influencing the wear resistance of these components. Combining forging and heat treatment enables precise control over the material’s microstructure. Modifying the heat treatment process, such as quenching and tempering, can enhance the material’s hardness and toughness while eliminating undesirable microstructures. This effectively improves the wear-resistant performance of the parts.

### 3.3. Service Environment

In the work process of agricultural machinery, abrasive wear predominates [50]. Interactions with soil, moisture, and other corrosive substances result in varying wear in key agricultural machinery components over extended service periods.

Under low-speed and heavy-load conditions, the contact stresses between gears rise, resulting in a localized contact stress concentration on parts’ surfaces. This heightened pressure and friction in the localized region can occur due to the difficulty of forming and maintaining a lubricant film during low-speed operation. Consequently, the friction between parts increases, and the dissipation of frictional heat becomes challenging [51]. In turn, it hinders the effective removal of metal particles and wear products from the surface of the parts, further exacerbating wear. In low-speed and heavy-load conditions, machinery operation often involves vibrations and impacts [52]. Mechanical components are susceptible to slight displacements and relative motions due to vibrations and impacts, accelerating the wear process.

Friction and wear caused by agricultural materials on the working parts of agricultural machinery are essential factors affecting their efficiency and energy consumption. Among them, the friction and wear of soil on soil-touching functional parts are the most typical. The wear of earth on soil-touching parts is a type of free-form abrasive wear [53]. The friction between the ground and the working components of agricultural machinery causes resistance to the machinery’s functioning. Resistance due to friction accounts for 20% to 30% of the total resistance to tillage [54]. Agricultural machinery implements such as rotary tillage knives and plowshares are the most widely used implements in agricultural machinery. Due to the long-term exposure to soil wear and the effects of alternating stress, processing defects within the substrate and other parts can easily lead to the formation of stress concentrations. This causes localized damage and accelerates the scraping speed of soil tillage components.

Agricultural machinery frequently encounters corrosive gases and liquids, including hydrogen sulfide (H_2_S) [55], ammonia (NH_3_) [56], fertilizer solutions, and pesticide solutions [57], during both operation and storage. The chemicals in corrosive gases and liquids are strongly acidic or alkaline and chemically react with the metals on the surface of the parts. This reaction leads to the release of metal ions and the corrosion of the metals [58]. This chemical reaction destroys the protective surface layer of the component, exposing it to oxygen, moisture, and other corrosive substances. This accelerates the corrosion and wear of the metallic material. When there is heterogeneity on the metal’s surface, such as physical defects and non-uniformity of the oxygen fraction, tiny cells are formed on the surface, which can lead to electrochemical corrosion [59]. This corrosive action leads to the localized dissolution and exfoliation of the metal surface. The products of decomposition and detachment can become abrasive when exposed to friction and movement, resulting in heightened friction and wear between components.

Soil, corrosive gases or liquids, and heavy loads at low speeds are three everyday service environments for farm machinery parts, and they have different effects on the wear resistance of machinery parts. The hardness and moisture content of the soil can increase friction and wear between a component and the soil, especially in dry and compacted soil conditions, making the piece more susceptible to significant wear. Corrosive gases or liquids can erode the part’s surface, leading to corrosion and material degradation, ultimately reducing the part’s durability. Heavy loads at low speeds can cause high contact pressure and friction between elements, increasing wear and fatigue fracture risk.

## 4. Improvement Measures for Wear Resistance of Key Components in Agricultural Machinery

With the continuous development of agricultural science and technology, more and more rural machinery is widely used in modern agricultural production. However, in the process of use, the wear in agricultural machinery components has become one of the main factors restricting the reliability and economy of agricultural machinery. To address this problem, most scholars continue exploring innovative ideas and implementing various improvements, such as mechanical parts structure optimization design, mechanical parts using wear-resistant materials, and mechanical parts surface preparation of wear-resistant coatings.

### 4.1. Structural Optimization Design

The failure of crucial components in agricultural machinery is not solely dependent on manufacturing materials and heat treatment processes but is also influenced by operational forces. By optimizing the design and structure of key components, the force distribution on wear parts can be improved, ensuring even wear and tear across the segments. In turn, this serves the purpose of extending the service life of vital agricultural machinery components.

Armadillidium vulgare, resembling an earthworm, possesses a smooth body surface capable of reducing soil adhesion [60]. Massah et al. [61] applied the body surface geometry of armadillos to a rotary tiller blade. The results demonstrated the effectiveness of the bionic rotary tiller blade in reducing the soil-cutting resistance, especially in moist soil conditions. Dung beetles’ front legs serve as practical bionic prototypes for soil-cutting tools. Zhang et al. [62] designed a gear based on the complex outer contour curves of the teeth at the end of dung beetles’ front limbs. This bionic gear reduced the traction force by 16.5%. A finite element analysis revealed that the bionic spiked teeth experienced the highest stress concentration at the point of contact with the soil. This improved the tool’s cutting performance, reducing the soil material’s adhesion and friction. Li et al. [63] also utilized the clawed toe of dung beetles as the foundation for designing and analyzing a bionic cutting disc. A comparison of the finite element simulation results indicated that the bionic disc exhibited 22.64% less stress than the standard disc, signifying its enhanced structural strength. Moreover, the soil stress of the bionic disc was 6.87% greater than that of the conventional disc, providing superior cutting ability. These advantages can be attributed to the clawed toe, which aids in directing broken soil backward along the ridge profile. Table 3 shows design examples of the bionic structural design of agricultural machinery parts.

While using bionics to optimize the design of essential components in agricultural machinery, it is also required to undertake in-depth research into soil organism movement characteristics. This is important to study the correlation between the motion features of an element and its parts, such as wear resistance and drag reduction, and to apply dynamic optimization techniques to the soil-touching components in agricultural machinery to further improve the wear resistance and reduce drag in these parts.

### 4.2. Abrasion-Resistant Material

Steel is favored for its outstanding mechanical properties, making it especially suitable for producing agricultural machinery and tools. Nevertheless, it is important to note that steel does have certain limitations, including vulnerability to wear and corrosion [65,66]. Specifically, when steel is exposed to highly abrasive environments or highly acidic agricultural soils, it can experience increased wear and corrosion, potentially failing agricultural machinery.

Ihor Koval et al. [67] researched the tribological properties of TiC-VC-WC/nanoWC-NiCr-cemented carbide alloys when sliding against alloy steels 52100 and 5040 at varying sliding velocities. The study demonstrated that the TiC-VC-WC/nanoWC-NiCr alloy exhibited favorable frictional properties. Specifically, the coefficient of friction for the friction pair with 52100 alloy steel was 0.46, whereas the coefficient of contention for the friction pair with AISI 5040 alloy steel was 0.62. Due to its exceptional wear resistance, this carbide can produce wear-resistant components for agricultural machinery, including plowshares, rotary tiller blades, and cutting elements for various agricultural equipment. The crawler plate, a part of crawler tractors, comes into direct contact with soil and gravel during operation. It experiences severe abrasive wear as hard abrasive materials in the ground become entrapped in its center hole, runway, and pin joints [68]. Tractor track plates were predominantly made from high manganese steel casting, but they lacked sufficient work-hardening during farmland operations, resulting in limited wear resistance. They were gradually replaced by materials like manganese steel (such as ZGMn8) and low-alloy martensitic cast steel (such as ZG30Mn2Si), in addition to some track plates made from ductile cast iron, ordinary carbon steel, or medium-carbon low-alloy steel through rolling processes [69].

At present, the critical wear-resistant components in agricultural machinery are mostly made with spring steel 60Si_2_Mn, with 65 Mn as the base material [70], and under force, the wear parts or edge parts are usually welded to a thickness varying from 1 to 2 mm, containing a hardened wear-resistant layer to extend their service life. At the same time, feed mill hammer blades are manufactured with rugged white cast iron, spade plow and rotary plow curved knives are manufactured with pearlite malleable cast iron, and pump impellers are manufactured with boron cast iron to ensure the necessary strength and toughness conditions to have a high degree of wear resistance.

### 4.3. Surface Strengthening

Surface strengthening is crucial for enhancing the wear resistance of agricultural machinery. Agricultural machinery often operates in challenging conditions, and surface wear is a primary source of damage [71]. Surface-strengthening technology can substantially increase mechanical parts’ surface hardness and wear resistance. Meanwhile, it also extends their lifespans, reduces maintenance expenses, and enhances the efficiency of agricultural production.

**Figure 12:**
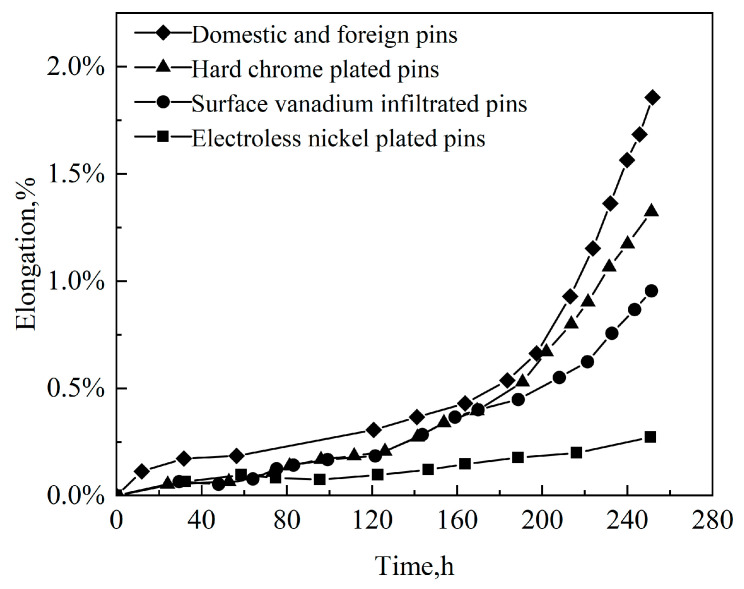
Elongation versus experiment time.

Wear-resistant coatings are applied to cutting tools’ surfaces to protect them and keep the correct edge shape. The current selection of wear-resistant materials in wear-resistant coatings mainly includes nickel–chromium alloys [72], tungsten carbide [73], nitride, ceramics [74,75], and alumina [76]. A. Salimi et al. [77] deposited thin copper films on ultrafine WC particles by the chemical ferrying method. Then, the nickel-based brazing material was mixed with a WC-Cu composite powder, and a high-temperature vacuum brazing coating was used to create a self-flowing alloy (NiCrBSi). The developed layer had a hardness of up to 1500 HV, sufficient for actual farm machinery operation. Yazici et al. [78] applied the gas carbonitriding method to a 30MnB5 plowshare after a field test revealed that, compared to a traditional heat treatment, gas carbonitriding reduced plowshare wear by 14.6% and reduced wear volume by 24.6% in the same area of farmland under cultivation. Nalbant et al. [79] applied TiN by physical vapor deposition, complex Ni by electrodeposition, and Cr by electroplating coatings to a plowshare’s surface in that order; after conducting field tests, it was determined that the TiN coatings exhibited superior wear resistance when compared to the hard Ni coatings and electroplated Cr coatings. Ye Bin et al. [80] conducted experiments on pin surfaces, including chemical nickel plating, hard chrome plating, vanadium infiltration, and various surface-strengthening treatments, and on three surface-strengthening chain segments with the same number of foreign links to form a chain mounted on both sides of an Ls910A closed force flow tester for a comparative wear test. This guarantees uniformity in the test conditions across all chain segments within the same chain. The chain was taken out every 10 h, and the length was gauged with an LSM5lA chain gauge. From Figure 12, after 160 h, there is a noticeable increase in the wear on pins from abroad. When it comes to the extent of wear and the duration it takes for additional wear to manifest, hard chrome-plated pins outperform foreign pins. Therefore, after the surface’s hard chrome plating treatment, the pin shows excellent wear resistance.

After a surface-strengthening treatment, the obtained coating thickness for agricultural machinery parts can be controlled between a few microns and a few millimeters. This effectively lowers the coating cost while significantly enhancing the hardness and fatigue strength, improving the wear resistance of crucial agricultural machinery components. Table 4 shows the standard hard-coating preparation methods and properties.

## 5. Challenges and Opportunities

### 5.1. Challenges

Research on the wear resistance of agricultural machinery has limitations. On the one hand, various factors such as the farmland environment, workload, and maintenance management can interfere with the wear resistance of agricultural machinery during actual use, leading to discrepancies between real-world performance and laboratory results. On the other hand, obtaining precise wear resistance test data can be time-consuming and costly, which may pose challenges for small and medium-sized enterprises. In addition, there are bottlenecks in the equipment and promotion of high-end agricultural machinery in China, mainly in the following areas.

(1) High-end agricultural machinery usually requires high-quality materials and advanced manufacturing processes to improve its performance and longevity to cope with the harsh agricultural working environment. However, these high-quality materials and processes increase manufacturing costs, resulting in expensive high-end agricultural machinery that is not conducive to its purchase and use by most farmers.
(2) China’s rural areas feature diverse agricultural practices with varying needs across regions and among farmers. Certain areas may require specific types of farm machinery or customization of machinery to align with distinct agricultural practices. This increases the complexity of developing and producing high-end agricultural machinery, limiting its large-scale promotion and popularization.
(3) Maintaining and repairing high-end farm machinery is costly, given their use of high-quality and often expensive parts and components. This can deter farmers from investing in these machines due to concerns about long-term maintenance costs.
(4) Rural China still lags behind developed countries regarding economic conditions and agricultural infrastructure, which hinders the extensive adoption of high-end farm machinery. Addressing this issue necessitates the government’s formulation of supportive policies to incentivize farmers to acquire high-end farm machinery. Furthermore, training and technical support should be provided to ensure effective utilization of such equipment in rural areas.

### 5.2. Opportunities

Research on the wear resistance of agricultural machinery holds significant importance for policymakers, practitioners, and the economic sector.

(1) Policymakers can benefit from this research by crafting intelligent and sustainable agricultural development policies. Encouraging agricultural machinery manufacturers to adopt more wear-resistant materials and manufacturing processes can enhance machinery performance and longevity, reducing farmers’ operational costs and boosting agricultural production efficiency. Moreover, government policies can incentivize farmers to utilize highly wear-resistant machinery through tax incentives or subsidies, fostering agricultural modernization and sustainable rural development.
(2) Practitioners, particularly agricultural machinery manufacturers, and maintenance personnel can leverage the findings of wear resistance research. This information can guide them in refining agricultural machinery’s design, material selection, and maintenance techniques, making their products more competitive and expanding their market share. Practitioners who develop more dependable and long-lasting farm machinery meet farmers’ needs and stimulate job creation and economic growth.
(3) The economic sector reaps rewards from agricultural machinery wear resistance research. Agriculture constitutes a vital segment of China’s economy, and improving the durability of agricultural machinery enhances production efficiency and output, ultimately increasing the supply of agricultural products. This, in turn, can lower agricultural product prices and elevate the living standards of both urban and rural residents. Additionally, the agricultural machinery manufacturing industry harbors substantial potential, and investment in research and development can drive technological innovation and industrial upgrading, catalyzing broader economic development.

In summary, the study of agricultural machinery wear resistance holds profound significance for policymakers, practitioners, and the economic sector. It facilitates agricultural modernization, augments rural productivity, enhances farmers’ livelihoods, and fosters sustainable agricultural and economic development.

## 6. Conclusions and Perspectives

### 6.1. Conclusions

This article systematically analyzes the research progress on the wear resistance of key components in agricultural machinery. The results are summarized as follows:

(1) Research into agricultural machinery wear resistance is progressively deepening to meet the rising demands of agricultural production. Scholars have unveiled the formation mechanism of wear and its key influencing factors in investigating wear mechanisms by exploring various wear types. Through continuous research on wear-resistant materials, scholars have provided essential theoretical and experimental foundations for enhancing the wear resistance of key components in agricultural machinery. Applying new wear-resistant technologies, such as nano-lubrication and computer-aided technology, has breathed new life into this field.
(2) The diversity of working environments for agricultural machinery results in varying wear behaviors under different conditions. Therefore, selecting materials is crucial and must precisely align with specific application scenarios and requirements. Furthermore, studies on material microstructure reveal that grain size significantly impacts material wear resistance, with finer grain sizes reducing the area of grain boundaries and thus enhancing wear resistance. The processing technology of parts directly affects their surface quality and wear resistance. Advanced processing techniques can improve a part’s surface finish and accuracy, reduce surface roughness, and minimize microscopic defects, enhancing wear resistance. During agricultural machinery operation, contact with various soil types, stones, plant residues, and other particles can cause surface wear on parts, ultimately reducing their wear resistance.
(3) To improve the wear resistance of key components in agricultural machinery, adopting a series of comprehensive measures is necessary. Firstly, it is possible to improve the shape and dimensions of components to adapt to high-wear environments through structural optimization. Increasing local thickness or altering surface shapes can help distribute force more evenly, reducing the amount of premature wear caused by concentrated stress. Secondly, the selection of appropriate wear-resistant materials is crucial. Materials like high-strength steel, hard alloys, and ceramic materials can significantly enhance the wear resistance of key components. Additionally, surface-strengthening treatments can increase the hardness of a metal surface layer, making it more resistant to wear and tear.
(4) There are limitations in the research on the wear resistance of agricultural machinery. Factors like the farmland environment and workload can affect test results. Moreover, traditional processing methods face technological bottlenecks, and adopting new technologies often requires significant capital investments. However, the research findings can guide policymakers in establishing policies and standards. Practitioners can also enhance their maintenance and management skills to extend machinery lifespans while improving the quality and competitiveness of agricultural machinery, thus facilitating agricultural modernization.

### 6.2. Perspectives

Studying the wear resistance of key components in agricultural machinery is an important research direction in agricultural machinery engineering. The development trends in this area mainly manifest in the following aspects.

(1) Advancements in materials science will play a significant role in driving research on agricultural machinery wear resistance. As materials science and technology continue to progress, the emergence of novel materials will open up new possibilities for enhancing the wear resistance of agricultural machinery. For instance, utilizing innovative materials like nanomaterials and composite materials will improve agricultural machinery’s wear resistance, reducing mechanical wear and damage and prolonging machinery’s service life.
(2) The advancement of surface-strengthening treatment technology will be a key focus in agricultural machinery wear resistance research. Implementing surface coating and modification technologies will significantly enhance agricultural machinery’s surface hardness and wear resistance. It will reduce the extent of wear on mechanical parts and extend machinery’s service life. Additionally, surface-strengthening treatment technology can enhance agricultural machinery’s functional capabilities, improving its adaptability to various operational environments and conditions.
(3) Using digital technology will play a significant role in agricultural machinery wear resistance research. With digital technology, real-time monitoring and analyses of wear and damage in agricultural machinery can be conducted. This enables the timely detection of machinery failures and wear, allowing for effective maintenance and repair measures to be implemented, ultimately extending the machinery’s service life. Moreover, digital technology can optimize agricultural machinery’s design and manufacturing processes, enhancing wear resistance and reliability.
(4) Green environmental protection requirements will be a significant focus in agricultural machinery wear resistance research. In the future, as society’s demands for environmental protection continue to rise, research in agricultural machinery wear resistance will increasingly prioritize green ecological protection concepts. This emphasis will drive efforts to minimize wear and damage to agricultural machinery, thereby reducing environmental pollution and harm.

## Figures and Tables

**Table 3:** Design examples of the bionic structural design of agricultural machinery parts [64].

Part Name	Bionic Principle	Physical Picture
Bionic design of plane bulldozing plate	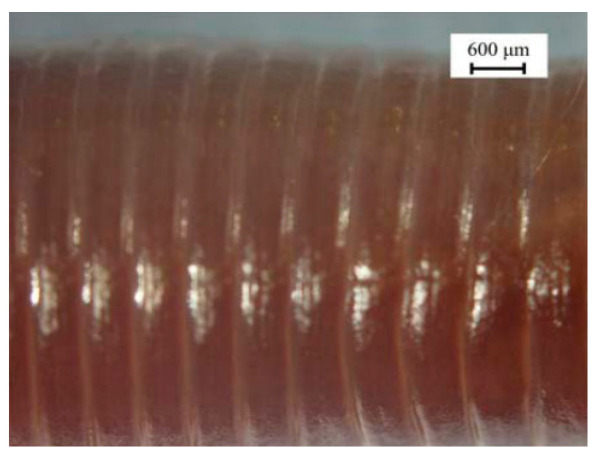	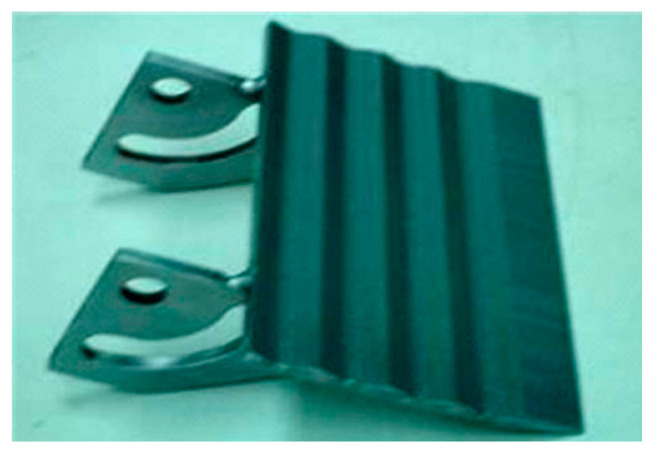
Bionic soil-engaging blade	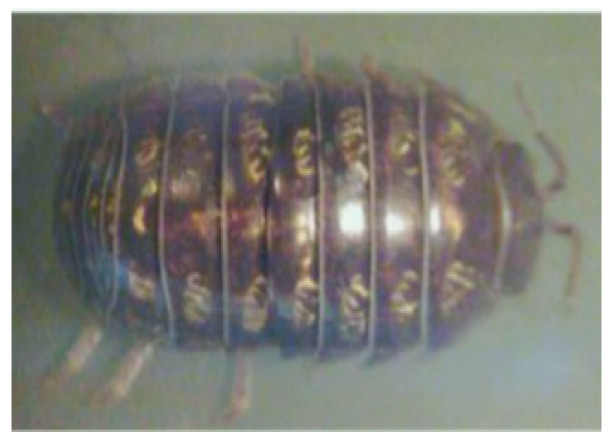	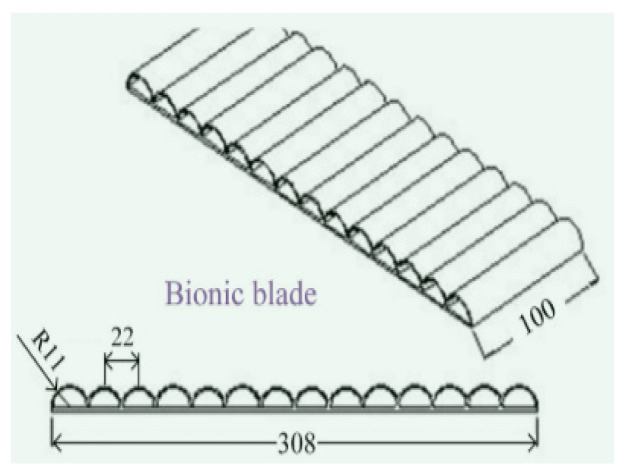
Bionic design of toothed wheel	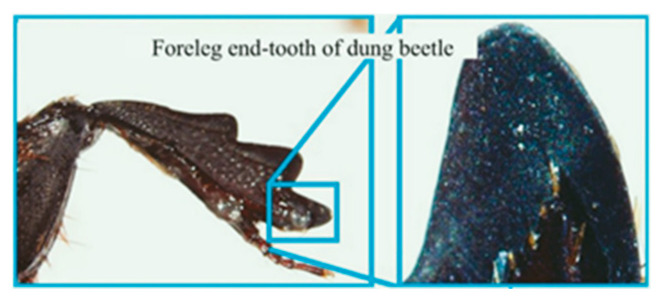	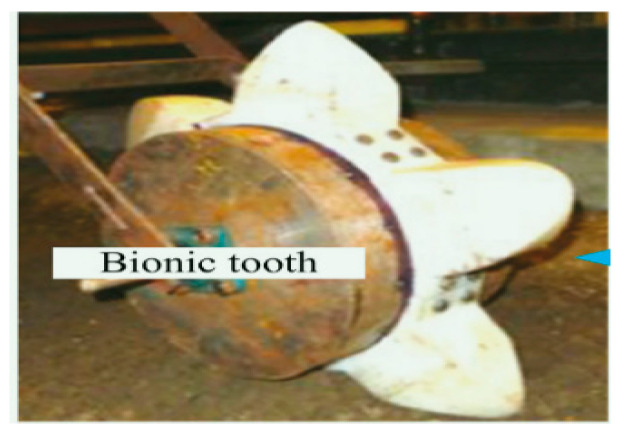

**Table 4:** Typical processes to prepare hard coatings.

Part Name/ Comparison Material	Stiff Coating and Preparation Method	References
Rotary cutter/EN-14B	Argon arc overlay coating	[81]
	Thermal spraying WC-10Co-4Cr	[82]
	Manual arc overlay of low-carbon- or stainless-steel-based wear-resistant coatings	[83]
	Thermal spray WC-Co- and Fe-based coatings	[84]
	Thermal spray WC-10Co-4Cr coating/powder metallurgy coating WC-5.7Co-0.3Cr	[85]
Rotary cutter /Fe-0.5C-0.9Mn-0.7Si	Supersonic flame spraying WC-Co coating	[86]

## Data Availability

No new data were created or analyzed in this study. Data sharing does not apply to this article.
